# Medico-economic comparison of two anticoagulant treatment strategies: Vitamin K antagonists vs. direct oral anticoagulants in older adults in nursing homes in France. The “MIKADO” study

**DOI:** 10.1371/journal.pone.0283604

**Published:** 2023-04-04

**Authors:** George Pisica–Donose, Matthieu Piccoli, Bastien Genet, Stéphane Bouee, Stefan Berechet, Ion Berechet, Antonin Dacasa Cortes, Sabri Atsamena, Catherine Bayle, Mihai Badescu, François Catelain, Lynda Kermeche, Isabelle Merlier, Sahondranirina Rakotoniary, Valérie Savin, Ariane Vidal, Jean-Sébastien Vidal, Olivier Hanon

**Affiliations:** 1 Medalice, Le Port Marly, France; 2 Memory Resource and Research Centre of de Paris-Broca-Ile de France, Hopital Broca, APHP, Paris, France; 3 EA 4468, Université de Paris, Paris, France; 4 CEMKA, Bourg-la-Reine, France; 5 SISPIA, Vincennes, France; 6 DomusVi, Suresnes, France; 7 EHPAD La Résidence de Ballancourt, Ballancourt-sur-Essonne, France; 8 EHPAD Péan, Paris, France; 9 EHPAD Fondation Condé, Chantilly, France; 10 EHPAD Richard, Conflans-Sainte-Honorine, France; 11 EHPAD La Gentilhommière, Boussy-Saint-Antoine, France; 12 EHPAD Centre Hospitalier de Brie Comte Robert, Brie-Comte-Robert, France; 13 EHPAD La Martinière, Sablé-sur-Sarthe, France; 14 EHPAD Centre Hospitalier de la Mauldre, Jouars-Pontchartrain, France; 15 EHPAD Résidence Baccarat, Baccarat, France; Brigham and Women’s Hospital, UNITED STATES

## Abstract

**Objectives:**

Currently, two classes of oral anticoagulants are available in nursing home residents: vitamin K antagonists (VKA) and direct oral anticoagulants (DOAC). DOACs have a higher net clinical benefit than VKAs but DOACs are about 10 times more expensive than VKAs. The objective of our study was to assess and compare the overall costs of anti-coagulant strategy (VKA or DOAC), i.e., including drugs, laboratory costs and time spent in human capital (nurses and medical time) in nursing homes in France.

**Methods:**

This was an observational, multicenter, prospective study including nine nursing homes in France. Among these nursing homes, 241 patients aged 75 years and older and treated with VKA (n = 140) or DOAC (n = 101) therapy accepted to participate in the study.

**Results:**

During the 3-month follow-up period, the adjusted mean costs per patient were higher for VKA than DOACs for nurse care (€327 (57) vs. €154 (56), p<.0001) for general practitioner care (€297 (91) vs. €204 (91), p = 0.02), for coordinating physicians care (€13 (7) vs. €5 (7), p < 0.07), for laboratory tests (€23 (5) vs. €5 (5), p<.0001), but were lower for drug costs (€8 (3) vs. €165 (3), p<.0001). The average overall cost for 3 months per patient was €668 (140) with VKA vs. €533 (139) with DOAC (p = 0.02).

**Conclusion:**

Our study showed that in nursing homes despite a higher drug cost, DOAC therapy is associated with a lower total cost and less time used by nurses and physicians for drug monitoring when compared to VKA.

## Introduction

Atrial fibrillation (AF) is a high-risk disease that affects more than 10% of people over 80 years [1]. The benefits of anticoagulant therapy have been widely demonstrated in interventional and observational studies. These benefits are even greater in older adults [2, 3].

Currently in France, more than 700,000 elderly people are living in nursing homes (NH) [4, 5] and approximately 14% of institutionalized old people are treated with oral anticoagulant drugs [6]. Thus, we can estimate that between 70,000 and 100,000 elderly people living in nursing homes are receiving anticoagulant therapy.

Two classes of oral anticoagulants are available: vitamin K antagonists (VKA) and direct oral anticoagulants (DOAC). Because the DOAC strategy has a higher net clinical benefit than VKA strategy, the European Society of Cardiology recommends the use of DOAC over VKA for stroke prevention in patients with atrial fibrillation [7, 8] and for venous thromboembolism prevention [9], even in older adults. One of the advantages of DOAC over VKA treatment is that it does not need any INR monitoring. On the other hand, serum creatinine measurements are necessary during the DOAC treatment surveillance.

The main argument against the use of DOACs is the high cost of the drugs, which in France is about 10 times higher than that of VKAs. However, when the costs of laboratory monitoring, which is mandatory for VKA, is included in the treatment costs, DOAC treatment costs are then only 6 times higher than that of VKA [10]. Moreover, the management of VKA treatment requires more medical and nursing time for INR monitoring and doses adjustment which is usually not taken into account in the studies [10].

In nursing homes, drug management is well monitored: general practitioners prescribe the drugs and follow the residents, coordinating physicians are responsible for case management and nurses distribute and monitor the residents’ medications. It is then possible to measure the time and the human capital spent for tasks like blood sample draw, laboratory result gathering, drug dose adjustment and interaction with other professionals (general practitioner, laboratory staff and pharmacists). Thus direct cost of anticoagulant therapy can be measured in nursing homes enabling the real-life assessment of the overall cost of DOAC and VKA treatments.

The objective of our study was to assess and compare the overall costs of anti-coagulant strategy (VKA or DOAC), i.e., including the drugs, laboratory costs and human capital in nursing homes in France.

## Materials and methods

MIKADO study (Medico-economic study comparing two anticoagulant treatment strategies (VKA vs. DOAC) in elderly patients living in nursing homes) was a real-world, observational, multicenter, pharmacoeconomic, prospective study from June 2017 to March 2019, including nine NH in France for a total of 1,572 residents.

While eight of those nine NH had specific management procedures for VKA therapy, none had the same procedures for DOAC. Seven NH had individual weekly drug preparation by pharmacist-in-charge and four had an in-house pharmacy.

Inclusion criteria were (1) residents living in NH (2) accepting to participate in the study, (3) aged 75 years and older, (4) already treated with VKA or DOAC therapy and (5) affected by one of the following conditions: (a) atrial fibrillation, (b) mechanical heart valve prosthesis, (c) pulmonary embolism and (d) deep vein thrombosis.

Treatment strategies and medications were chosen by the patients’ general practitioners. It was a real-world study and the study board had no influence on them.

The study was submitted and approved by the French Ministry of Higher Education, Research and Innovation—Advisory Committee on the Processing of Information in Health Research (CCTIRS).

Each nursing home has an internal ethics committee: “The Social Life Committee” which approved the study before it began. Participants and their families were informed in writing and by the coordinating physician that some demographic and clinical data obtained from patient’s medical file were to be used for the study. However no signed written consent was sought since French laws (loi n° 2012–300, March 5 2012, Décret n° 2016–1537) and research rules do not require written consent for non-interventional studies but only information about the study and non-opposition of the participants and/or their families.

All data were anonymized and managed according to the French data protection authority (CNIL).

Time spent for the management of the anticoagulant therapy was assessed on a daily base by the healthcare team (coordinating physicians and nurses) of each NH participating in the study for 3 months and self-recorded daily on a dedicated file.

Time spent for the management of the anticoagulant therapy included medication delivery, blood draw and lab exams for INR and serum creatinine measurement, time spend to gather the biological results and to transmit them to the GPs, time spent by the GPs to check the INRs, serum creatinine measurements and to adjust the dosage and time spent to manage clinical events related to anticoagulant therapy like bleedings, medical consultations or death.

Time was self-reported in minutes per day and was set to 0 if no time was spent for a given patient on a given day for anticoagulation management.

During the 3 months of follow up, 9 coordinating physicians and 97 nurses in charge of the daily recording fed more than 530,000 pieces of information on dedicated files that included the overall time spent for management of the anticoagulant therapy.

To compare the overall cost of each strategy (VKA vs. DOAC), we added the disease management costs (anticoagulant drugs, laboratory analyses as INR and estimated with glomerular filtration (eGFR)), to the nurse and medical staff costs for this specific charge (by multiplying the hourly wage by the time spent for each anticoagulant treatment).

Major bleeding was defined according to the International Society on Thrombosis and Haemostasis: clinically overt bleeding associated with any of the following: (1) death; (2) involvement of a critical anatomical site (intracranial, spinal, ocular, pericardial, retroperitoneal, articular or intramuscular with compartment syndrome); (3) drop in hemoglobin concentration ≥ 20 g/L and (4) transfusion ≥ 2 units of whole blood or red blood cells [11] Hemorrhages that did not meet major bleeding criteria were categorized as non-major bleedings.

Demographic and clinical data and comorbidity were obtained from patients’ file and medical history and clinical examination by the coordinating physician of each structure.

eGFR was estimated by Cockcroft and Gault formula. Thromboembolic risk was assessed by CHA_2_DS_2_-Vasc score [12] and bleeding risk by HAS-BLED score [13].

The analysis was carried out from the perspective of the French Health Insurance (FHI) and all costs are given in euros. In all nursing homes in France, the medical costs (such as medical consultations, nurses’ salaries, laboratory cost, medication costs) are fully covered by FHI. In this study, the cost estimation was made by adding staff costs (nurses and physicians), specific lab tests (INRs and eGFR) and drug costs. Nurses and coordinating physicians are always employees of the NH and their costs are based on mean wages and time spent for management of anticoagulant therapy [14]. General practitioners operate in NH as private practice physician for prescription and their average hourly income are estimated by statistic publication of French Health Insurance data [15, 16]. Drug costs were taken from the French public drug database [17] and an average cost per patient per month calculated from the recommended dose in the summary of product characteristics of each product and the costs per daily dose. One participant in the DOAC arm had missing information about DOAC drug used. We imputed an average DOAC cost based on the cost of each DOAC weighted by the frequency of each DOAC type in our study.

Only the costs of VKA and DOAC were taken into account in this study. We did not take into account the costs of other medications.

Costs of laboratory tests are based on the FHI quotation [18]. Time used to manage events like non-major bleedings was accounted in the overall time spent by nurses and GPs.

All costs used for analysis are shown in Table 1.

**Table 1:** Cost estimations.

Staff costs	Cost per hour
Nurse	€33.40
General practitioner	€78.90
Coordinating physician	€68.80
DOAC costs	Cost per month
Rivaroxaban	€53.40
Apixaban	€62.40
Dabigatran	€57.20
VKA costs	Cost per month
Warfarin	€5.39
Fluindione	€2.91
Acenocoumarol	€2.17
Laboratory test costs	Cost per test
International normalized ratio	€5.40
Estimated glomerular filtration rate	€2.40

DOAC, direct oral anticoagulants; VKA, vitamin K antagonists

General characteristics of the study population, costs and events were analyzed in the whole sample and according to the use of DOAC or VKA. Categorical variables are presented as percentage and counts (% (N)), continuous variables as mean and standard deviation (M (SD)) and comparison were made with χ^2^ tests and T-tests respectively.

We then performed a generalized linear model (GLM) with total costs as dependent variable and treatment group as independent variable with adjustment for age, sex and cardiovascular conditions (i.e., hypertension, heart failure, ischemic heart disease, history of stroke or TIA), and variables of the geriatric syndrome (i.e., depression, dementia and falls) and indication of anticoagulation (i.e., atrial fibrillation, deep vein thrombosis, pulmonary embolism and heart valve prosthesis).

Because costs were not normally distributed, we also analyzed overall costs between VKA and DOAC with a GLM model with gamma distribution and log link. The different costs were not analyzed separately because some participants had no cost in one or more individual costs and GLM model with gamma distribution and log link can only deal with variable with no-null values.

We also conducted sensitivity analysis with exclusion of participants with heart valve prosthesis indication of anticoagulant.

Statistical analyses were performed with R (R Foundation for Statistical Computing, Vienna, Austria. URL https://www.R-project.org/). A p value < 0.05 was used for statistical significance.

### Analytical sample

Of the 1,572 residents 249 patients received oral anticoagulant therapy and met the inclusion criteria; one patient switched from VKA to DOAC during the 3 months follow-up and was therefore excluded; 7 patients refused to continue, so 241 patients (140 VKA and 101 DOAC) were included in the analysis.

## Results

Patients’ characteristics (Table 2) show high prevalence of very old patients (mean age = 87.5 (6.2) years old) with severe disability (mean activities of daily living (ADL) = 2.65 (1.55), high number of comorbidities (34.8% of dementia, 48.8% of malnutrition (serum albumin < 35 g/l), 28.9% of depression, 24.9% of anemia (according to WHO criteria: hemoglobin < 130 g/l in men and < 120 g/l in women [19]), 20.3% of ischemic heart disease, 20.7% of heart failure, 22.0% of falls, 14.1% of severe renal insufficiency (eGFR < 30ml/min) and polypharmacy (mean number of other treatments than anticoagulants = 7.9 (2.8)).

**Table 2:** Patients’ characteristics in the whole sample and according to anticoagulation strategy.

General characteristics, % (n)	Whole sample	VKA	DOAC	Standardized difference	p*
	N = 241	N = 140	N = 101		
Age, M (SD)	87.5 (6.2)	87.9 (6.5)	87.1 (5.9)	-0.175	0.35
Age ≥ 85 years old	0.734 (0.443)	0.721 (0.450)	0.752 (0.434)	0.068	0.59
Women	75.1 (181)	70.0 (98)	82.2 (83)	0.245	0.04
Weight, M (SD)	68.5 (15.9)	68.9 (15.3)	67.9 (16.7)	-0.087	0.64
BMI, M (SD)	28.5 (14.1)	26.7 (5.8)	31.3 (21.2)	0.418	0.03
Indication for anticoagulant therapy					
Atrial fibrillation	92.1 (222)	92.9 (130)	91.1 (92)	-0.069	0.79
Deep vein thrombosis	4.56 (11)	2.14 (3)	7.92 (8)	0.208	0.07
Heart valve prosthesis	3.32 (8)	5.00 (7)	0.990 (1)	-0.413	0.18
Pulmonary embolism	4.56 (11)	2.14 (3)	7.92 (8)	0.208	0.07
Comorbidity					
Hypertension	78.0 (188)	82.1 (115)	72.3 (73)	-0.275	0.10
Heart failure	20.7 (49)	22.5 (31)	18.2 (18)	-0.114	0.52
Ischemic heart disease	20.3 (48)	25.5 (35)	13.0 (13)	-0.403	0.03
Anemia	24.9 (59)	23.4 (32)	27.0 (27)	0.080	0.63
Diabetes	12.0 (29)	13.6 (19)	9.90 (10)	-0.125	0.51
Falls	22.0 (35)	17.4 (16)	28.4 (19)	0.227	0.15
Depression	28.9 (69)	24.6 (34)	34.7 (35)	0.196	0.12
Dementia	34.8 (81)	26.8 (37)	46.3 (44)	0.335	0.003
BPSD	22.8 (55)	20.7 (29)	25.7 (26)	0.111	0.45
History of stroke or TIA	16.4 (39)	12.3 (17)	22.0 (22)	0.220	0.07
Number of drugs prescribed	7.89 (2.80)	7.83 (2.64)	7.97 (3.00)	0.072	0.75
Biological characteristics					
Serum creatinine (μmol/L), M (SD)	78.1 (35.2)	83.8 (43.1)	71.3 (20.6)	-0.520	0.03
eGFR (mL/min), M (SD)	55.7 (25.9)	54.0 (25.6)	57.8 (26.2)	0.146	0.38
Hemorrhagic and thrombotic scores					
HAS-BLED score, M (SD)	2.42 (1.45)	2.43 (1.47)	2.41 (1.42)	-0.022	0.91
CHA_2_DS_2_-Vasc score, M (SD)	3.90 (1.47)	3.80 (1.42)	4.04 (1.53)	0.230	0.21

% (n), percentage (count); M (SD), mean (standard deviation); VKA, vitamin K antagonists; DOAC, direct oral anticoagulants; BMI, body mass index in kg/m^2^; BPSD, behavioral and psychological symptoms of dementia; TIA, transient ischemic attack; eGFR, glomerular filtration rate estimated with Cockcroft formula; ADL, activities of daily living; MMSE, mini mental state examination.

* Comparison with T-tests or χ².

Indication for anticoagulant therapy was mainly for atrial fibrillation (92.1% of total patients, (92.9% of VKA patients vs. 91.1% of DOAC patients), p = 0.79).

Characteristics of the patients with exclusion of those with mechanical heart valves (N = 8) are shown in S1 Table.

### Treatment details

Among 140 patients that were receiving VKA, 54 (38.6%) were receiving warfarin, 78 (55.6%) fluindione, 8 (5.7%) acenocoumarol. Among 101 patients that were receiving DOAC, 57 (56.4%) were receiving rivaroxaban, 32 (31.7%) apixaban, 11 (10.9%) dabigatran and 1 (1.0%) patient received a DOAC without details.

### Primary outcome—costs analysis

During the 3-month follow-up period, time spent to monitor anticoagulant treatments was daily collected for nurses and physicians (general practitioners and coordinating physicians).

The time spent by nurses for anticoagulant treatment monitoring (adding time for drug management, preparation, administration and blood tests) for the 3-month study time was 483 (397) minutes per patient treated with VKA vs. 188 (150) minutes per patient treated with DOAC, p <0001).

The total nurse cost per patient for 3 months was estimated to be €269.14 (221.18) per patient treated with VKA vs. €104.82 (83.38) per patient treated with DOAC (p<.0001).

Mean time spent by general practitioners to prescribe, manage and monitor anticoagulant treatments during the 3 months of the study was 151 (259) minutes per patient for VKA vs. 92 (146) minutes per patient for DOAC (p = 0.03), corresponding to a mean staff cost for general practitioner of €198.44 (340.85) per patient for VKA vs. €120.64 (191.97) per patient for DOAC, p = 0.03).

Time spent by coordinating physicians on anticoagulant treatment monitoring was (11 (24) minutes for patients treated with VKA vs. 2 (8) minutes for patients treated with DOAC, p <.0001), corresponding to a mean staff cost of €12.63 (27.06) per patient treated with VKA vs. €2.24 (8.92) per patient treated with DOAC (p < 0.0001).

During the follow-up period, we recorded 498 INR tests for the VKA patients with a mean of 3.56 (3.42) INR per patient treated with VKA patient and 21.4% had an INR > 3 and 27.0% an INR < 2 which are the same as national data recorded at 450 medical analysis laboratories [16]. No INR was performed in DOAC group. Serum creatinine monitoring was done for both VKA (10.0%) and DOAC strategy (43.6%). Globally mean costs for laboratory tests were higher for VKA strategy than DOAC strategy €19.57 (18.73) vs. €1.57 (2.41), p <.0001). In the VKA strategy, INR monitoring cost €19.21 (18.47) per patient and serum creatinine €0.36 (1.35) per patient. In DOAC strategy, laboratory costs were only for serum creatinine, €1.57 (2.41).

Drugs costs were calculated according to drug price and an average dose per patient. In the VKA group we recorded warfarin, fluindione and acenocoumarol and in DOAC group rivaroxaban, apixaban and dabigatran. During the 3 months follow-up period the mean drug costs was €11.35 (3.50) per patient treated with VKA vs. €170.43 (11.35) per patient treated with DOAC (p <.0001).

In total, when adding costs for nurses, physicians (general practitioners and coordinating physicians), laboratory tests and drug costs, the average total cost for 3 months was €511.13 (532.43) per patient treated with VKA vs. €399.70 (258.76) per patient treated with DOAC strategy, which corresponds to a difference of €111.43 per patient (p = 0.03) in favor of DOAC strategy.

**Fig 1:**
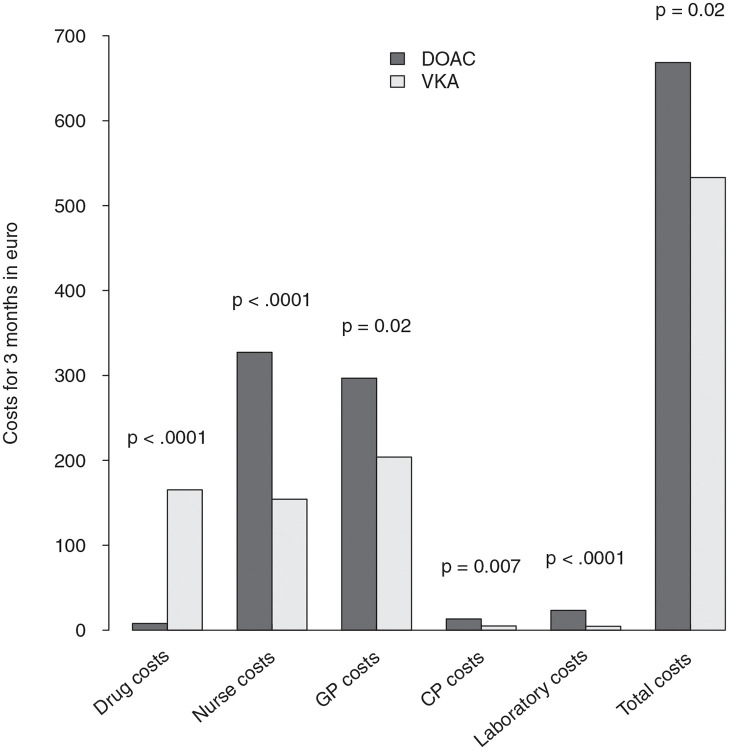
Detailed and overall costs of DOAC and VKA treatments for 3 months. VKA, vitamin K antagonists; DOAC, direct oral anticoagulants; GP, General practitioner; CP, coordinating physician.

Table 3 and Fig 1 summarize the costs and time adjusted for age, sex, cardiovascular disease (i.e., hypertension, heart failure, coronary heart disease, stroke and TIA), dementia, depression, anticoagulant indication (i.e., atrial fibrillation, pulmonary embolism, deep vein thrombosis and heart valve prosthesis).

**Table 3:** Costs and time spent by nurses and physicians during 3 months according to DOAC and VKA treatments.

Adjusted costs and time per 3 months in euro and minutes, AM (SE)	VKA	DOAC	p
	N = 140	N = 101	
Drug cost	€8 (3)	€165 (3)	<.0001
Time per patient spent by nurses	587 (102)	277 (101)	<.0001
Nurses cost	€327 (57)	€154 (56)	<.0001
Time per patient spent by GP	226 (69)	155 (69)	0.02
GP cost	€297 (91)	€204 (91)	0.02
Time per patient spent by CP	11.5 (6.1)	4.40 (6.05)	0.007
CP cost	€13 (7)	€5 (7)	0.007
Biology cost	€23 (5)	€5 (5)	<.0001
Total costs	€668 (140)	€533 (139)	0.02

AM (SE), mean (standard error) adjusted for age, sex, cardiovascular disease (i.e., hypertension, heart failure, coronary heart disease, stroke and TIA), dementia, depression, anticoagulant indication (i.e., atrial fibrillation, pulmonary embolism, deep vein thrombosis and heart valve prosthesis); VKA, vitamin K antagonists; DOAC, direct oral anticoagulants; GP, general practitioner; CP, coordinating physician.

During the 3-month follow-up period, the adjusted mean costs per patient were higher for VKA than DOACs for nurses care (€327 (57) vs. €154 (56), p<.0001) for general practitioner care (€297 (91) vs. €204 (91), p = 0.02), for coordinating physicians care (€13 (7) vs. €5 (7), p < 0.007), for laboratory tests (€23 (5) vs. €5 (5), p <.0001), but were lower for drug costs (€8 (3) vs. €165 (3), p<.0001).

The total adjusted cost for 3 months was €668 (140) per patient treated with VKA vs. €533 (139) per patient treated with DOAC strategy (p = 0.02). The cost of VKA per patient for 3 months was €135 higher than the cost of DOAC.

When patients with mechanical heart valve were excluded, the cost of VKA per patient for 3 months was €138, p = 0.03 higher than the cost of DOAC (S2 Table).

Because total cost variable was not normally distributed, we ran a GLM with gamma distribution and log link with the same adjustment (i.e., age, sex, cardiovascular disease (i.e., hypertension, heart failure, coronary heart disease, stroke and TIA), dementia, depression, anticoagulant indication (i.e., atrial fibrillation, pulmonary embolism, deep vein thrombosis and heart valve prosthesis)). In this model, costs of VKA was still significantly higher than costs for DOAC, p = 0.02, (see S3 Table).

When patients with mechanical heart valve were excluded, the cost of VKA was still significantly higher than costs for DOAC, p = 0.01.

Few adverse events were reported during the follow-up period. Non-major bleedings did not require any hospitalization or visit to the emergency room and were managed within the nursing homes and the time and costs used to manage these non-major bleedings were accounted in the nurses’, the GPs’ and the coordinating physicians’ time and costs. One major bleedings occurred in the VKA group and there was no stroke.

## Discussion

Our study measured the direct costs of anticoagulant treatment in real life of French nursing homes, taking into account the time spend by health professionals involved in management of these treatments and the costs of laboratory tests and drug costs. While our study was a cohort study, it did not analyze the clinical outcomes of these drugs.

In our population of institutionalized older adults, the average total cost for 3 months was significantly lower with DOAC treatment compared with VKA (difference of €111 per patient, p = 0.03). This result remained significant even after adjustment for confounding factors including age, sex, cardiovascular conditions and comorbidity (€135 per patient, p = 0.02), and after using a GLM with gamma distribution and log link to take in account that the overall cost was not normally distributed.

Despite a higher drug cost, the DOAC therapy is less time consuming for health professionals (nurses, general practitioners and coordinating physicians) and less expensive in laboratory testing. This aspect appears very important for nursing home’s management, because the number of physicians and nurses are low in these settings, thus above economic aspect, the time saved can be used for the management of other patients and a better quality of life.

Unlike other usual medico-economic studies, we took into account real costs and expenses for anticoagulant treatment, we measured the time really spent for patients so our results are closer to reality than other studies based on theoretical models and conducted to assess the cost-effectiveness of some DOAC vs. warfarin or systematic reviews or other analysis conducted with multistate Markov model [20].

Few studies have been prospectively conducted with daily feeding of time spent by physician and nurses for management of anticoagulant therapy. During the 3 months of follow up, nurses and physicians recorded more than 530,000 pieces of information on dedicated files including overall time spent for management of anticoagulant therapy.

Control studies have demonstrated that stroke and intra cranial bleeding are significantly reduced in in patients 75 years and older treated by DOAC compared to VKA [20, 21]. This suggests that the overall medico economic benefit might be more important with DOAC considering the prolonged treatment duration that increases the occurrence of intracranial hemorrhages or stroke.

Nurses and GPs in NH are difficult to hire and their time is very important for the quality of care in these structures and to save it can improve the quality of life of their residents. As an example, 10 to 15 residents in a NH using only DOAC treatments instead of VKA would save time worth an equivalent of five to eight weeks of nurse time per year.

### Limitations

Even if our study was not randomized, patients in both groups at baseline were not significantly different in age, HAS-BLED, CHA_2_DS_2_-Vasc scores and comorbidities. Dementia, stroke/TIA and disability were more frequent in the DOAC group than VKA group but this cannot explain the lower time spent for DOAC patient (demented or patient with disability usually require more time than non-demented patients).

All patients included in the study were already treated with anticoagulants, but we do not have the time lag between the onset of anticoagulation treatment and the inclusion in the study. Differential duration of disease between VKA and DOAC treated patients might have resulted in different disease severity and explained the higher cost with VKA. Women composed 75% of our whole sample, which is consistent with the proportion of women in NH in France. They took significantly more often DOAC than men. We do not have any explanation for this finding that might be a random finding.

The study did not have enough power to compare specific DOAC (i.e., Rivaroxaban, Apixaban, Dabigatran) to warfarin.

There also might have been some time spent not reported by the nurses and coordinating physician. However there is also no indication that this would happen more often in the DOAC group than VKA group. Furthermore because VKA requires more interventions form nurses and GP than DOAC, any oversight would have favor VKA over DOAC.

The study only reflects the costs incurred in NHs and we do not have information on costs outside of nursing homes. However, during the 3-month follow-up only one major bleeding occurred and required a visit to the emergency room. This major bleeding occurred in the VKA group. In our study, costs of VKA management was higher than costs of DOAC management even without taking into account the costs of the major bleeding that occurred in the VKA group. Moreover, no stroke occurred during the 3-month follow-up. Most routine-care (biology and GPs’ cares) were managed in the NHs and taken into account for the cost analysis and only few other cares related to anticoagulation occurred outside of the NHs.

Finally, patients were only followed up for 3 months and our results might not be generalized to longer periods.

## Conclusions

Our study showed that in nursing homes despite a higher drug cost, DOAC therapy is associated with a lower total cost and less time used by nurses and physicians for drug monitoring when compared to VKA. Using DOAC therapy, when appropriate, could enable health care professionals to spend more time to other tasks, for example taking care of more patients, optimizing biological monitoring or other prescriptions, as well as taking care of quality of life in all its aspects.

## Supporting information

S1 Table Patients’ characteristics, patients with heart valve prosthesis excluded. (PDF)

S2 Table Costs and time spent by nurses and physicians during 3 months according to DOAC and VKA treatments, patients with heart valve prosthesis excluded. (PDF)

S3 Table GLM model with total costs as dependent variable and anticoagulant treatment as independent variable with other covariates using gamma distribution and log-link. (PDF)
