# Correction: Medico-economic comparison of two anticoagulant treatment strategies: Vitamin K antagonists vs. direct oral anticoagulants in older adults in nursing homes in France. The “MIKADO” study

**DOI:** 10.1371/journal.pone.0306377

**Published:** 2024-06-27

**Authors:** George Pisica–Donose, Matthieu Piccoli, Bastien Genet, Stéphane Bouee, Stefan Berechet, Ion Berechet, Antonin Dacasa Cortes, Sabri Atsamena, Catherine Bayle, Mihai Badescu, François Catelain, Lynda Kermeche, Isabelle Merlier, Sahondranirina Rakotoniary, Valérie Savin, Ariane Vidal, Jean-Sébastien Vidal, Olivier Hanon

In Fig 1, the color legend of bar graph is swapped. The dark gray corresponds to VKA while the white is for DOAC. Please see the correct Fig 1 here.

**Fig 1:**
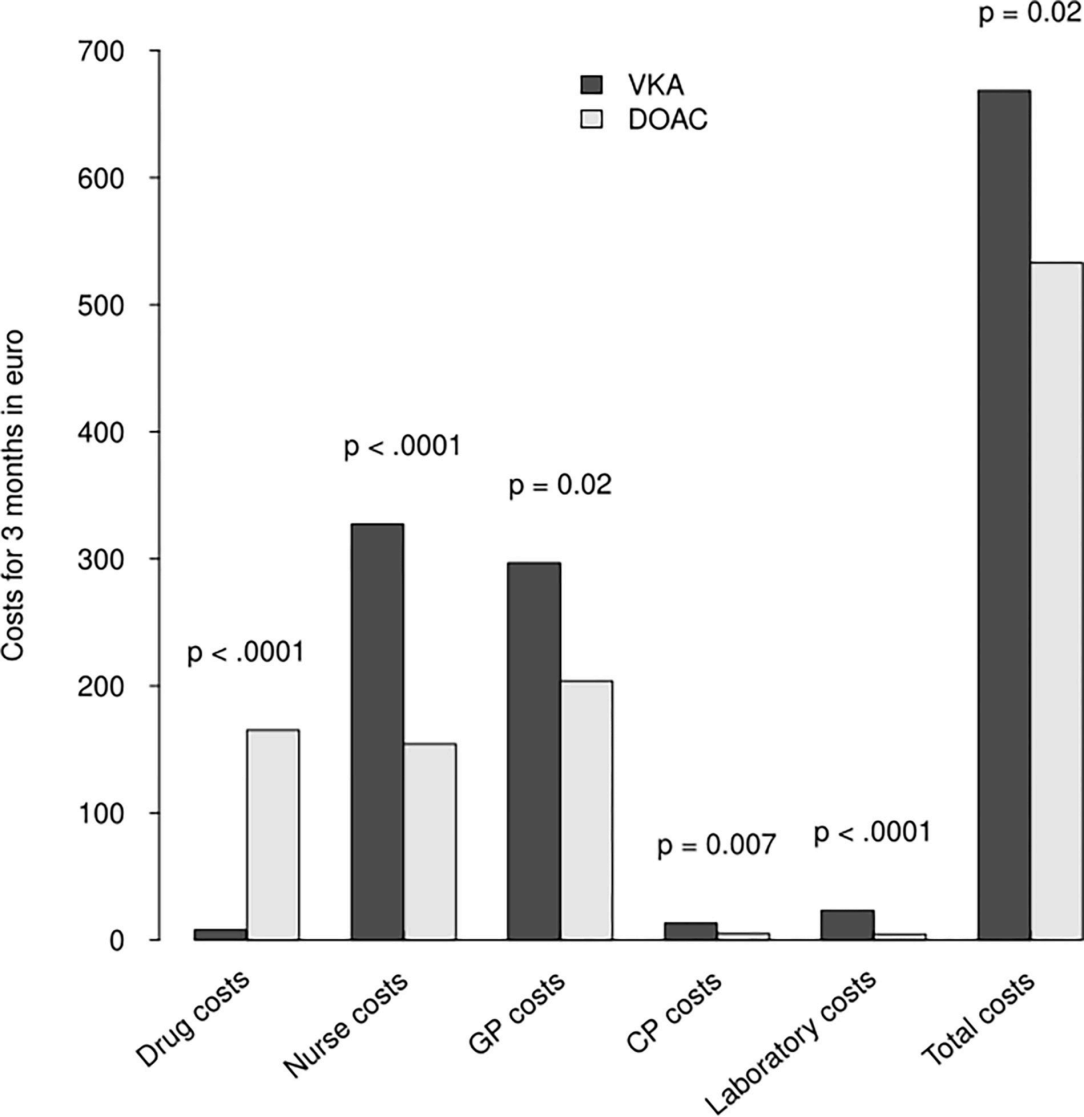
Detailed and overall costs of DOAC and VKA treatments for 3 months. VKA, vitamin K antagonists; DOAC, direct oral anticoagulants; GP, General practitioner; CP, coordinating physician.
