# Supplementary material for: Medico-economic comparison of two anticoagulant treatment strategies: Vitamin K antagonists vs. direct oral anticoagulants in older adults in nursing homes in France. The “MIKADO” study
Source: PLoS One. 2023 Apr 4;18(4):e0283604. doi: 10.1371/journal.pone.0283604 (PMC10072791; doi:10.1371/journal.pone.0283604)
Supplement: S1 Table — (PDF) [file pone.0283604.s001.pdf]

S1 table. Patients' characteristics, patients with heart valve prosthesis excluded.

General characteristics, % (n)	Whole sample	VKA	DOAC	Standardized difference	p1
	N=233	N=133	N=100		
Age, M (SD)	87.6 (6.3)	88.0 (6.5)	87.0 (5.9)	-0.167	0.21
Age ≥ 85 years old	73.8 (172)	72.9 (97)	75.0 (75)	0.047	0.84
Women	75.1 (175)	69.9 (93)	82.0 (82)	0.281	0.05
Weight, M (SD)	68.7 (16.0)	69.2 (15.4)	68.0 (16.8)	-0.075	0.57
BMI, M (SD)	28.7 (14.3)	26.9 (5.8)	31.4 (21.3)	0.317	0.04
Indication for anticoagulant therapy					
Atrial fibrillation	94.0 (219)	96.2 (128)	91.0 (91)	-0.221	0.17
Deep vein thrombosis	4.72 (11)	2.26 (3)	8.00 (8)	0.272	0.08
Pulmonary embolism	4.72 (11)	2.26 (3)	8.00 (8)	0.272	0.08
Comorbidity					
Hypertension	78.5 (183)	83.5 (111)	72.0 (72)	-0.281	0.05
Heart failure	19.2 (44)	19.8 (26)	18.4 (18)	-0.037	0.91
Ischemic heart disease	19.6 (45)	25.2 (33)	12.1 (12)	-0.332	0.02
Anemia	24.0 (55)	21.5 (28)	27.3 (27)	0.134	0.40
Diabetes	12.0 (28)	13.5 (18)	10.00 (10)	-0.108	0.54
Falls	23.2 (35)	18.8 (16)	28.8 (19)	0.236	0.21
Depression	28.1 (65)	23.7 (31)	34.0 (34)	0.230	0.11
Dementia	34.7 (78)	26.7 (35)	45.7 (43)	0.406	0.005
BPSD	22.7 (53)	20.3 (27)	26.0 (26)	0.136	0.38
History of stroke or TIA	16.1 (37)	12.2 (16)	21.2 (21)	0.246	0.10
Number of drugs prescribed	7.88 (2.75)	7.75 (2.52)	8.01 (3.00)	0.095	0.56
Biological characteristics					
Serum creatinine (µmol/L), M (SD)	78.2 (35.7)	84.6 (44.2)	71.0 (20.6)	-0.385	0.02
eGFR (mL/min), M (SD)	55.9 (26.2)	54.0 (26.2)	58.2 (26.2)	0.161	0.34
Hemorrhagic and thrombotic scores					
HAS-BLED score, M (SD)	2.40 (1.46)	2.40 (1.50)	2.40 (1.43)	0.001	0.99
CHA_2DS_2-Vasc score, M (SD)	3.88 (1.48)	3.77 (1.44)	4.02 (1.52)	0.166	0.21

% (n), percentage (count); M (SD), mean (standard deviation); VKA, vitamin K antagonists; DOAC, direct oral anticoagulants; BMI, body mass index in kg/m²; BPSD, behavioral and psychological symptoms of dementia; TIA, transient ischemic attack; eGFR, glomerular filtration rate estimated with Cockcroft formula; ADL, activities of daily living; MMSE, mini mental state examination.

* Comparison with T-tests or χ^2.
