# Supplementary material for: Medico-economic comparison of two anticoagulant treatment strategies: Vitamin K antagonists vs. direct oral anticoagulants in older adults in nursing homes in France. The “MIKADO” study
Source: PLoS One. 2023 Apr 4;18(4):e0283604. doi: 10.1371/journal.pone.0283604 (PMC10072791; doi:10.1371/journal.pone.0283604)
Supplement: S2 Table — (PDF) [file pone.0283604.s002.pdf]

S2 Table. Costs and time spent by nurses and physicians during 3 months according to DOAC and VKA treatments, patients with heart valve prosthesis excluded.

Costs and time per 3 months in euro and minutes, M (SD)	VKA N=133	DOAC N=100	p*
Drug cost	€10.24 (2.25)	€167.63 (2.20)	<.0001
Time per patient spent by nurses	585 (81)	268 (79)	<.0001
Nurses cost	€325.71 (45.14)	€149.29 (44.11)	<.0001
Time per patient spent by GP	263 (55)	193 (54)	0.02
GP cost	€345.46 (72.96)	€254.26 (71.29)	0.02
Time per patient spent by CP	11.1 (4.6)	3.32 (4.52)	0.002
CP cost	€12.72 (5.30)	€3.81 (5.18)	0.002
Biology cost	€24.33 (3.81)	€5.32 (3.72)	<.0001
Total costs	€718.47 (111.72)	€580.30 (109.08)	0.02

AM (SE), mean (standard error) adjusted for age, sex, cardiovascular disease (i.e., hypertension, heart failure, coronary heart disease, stroke and TIA), dementia, depression, anticoagulant indication (i.e., atrial fibrillation, pulmonary embolism, deep vein thrombosis and heart valve prosthesis); VKA, vitamin K antagonists; DOAC, direct oral anticoagulants; GP, general practitioner; CP, coordinating physician.
