# Supplementary material for: Medico-economic comparison of two anticoagulant treatment strategies: Vitamin K antagonists vs. direct oral anticoagulants in older adults in nursing homes in France. The “MIKADO” study
Source: PLoS One. 2023 Apr 4;18(4):e0283604. doi: 10.1371/journal.pone.0283604 (PMC10072791; doi:10.1371/journal.pone.0283604)
Supplement: S3 Table — (PDF) [file pone.0283604.s003.pdf]

S3 Table: GLM model with total costs as dependent variable and anticoagulant treatment as independent variable with other covariates using gamma distribution and log-link.

Covariates	Estimate (SE)	t value	p
VKA treatment	0.268 (0.118)	2.266	0.02
Age	-0.011 (0.009)	-1.168	0.24
Women	0.025 (0.131)	0.195	0.85
Dementia	0.087 (0.133)	0.658	0.51
Depression	0.536 (0.146)	3.674	0.0003
Cardiac condition			
Hypertension	-0.076 (0.140)	-0.54	0.59
Heart failure	0.087 (0.148)	0.587	0.56
Coronary artery disease	-0.017 (0.148)	-0.112	0.91
Stroke and TIA	0.081 (0.168)	0.48	0.63
Anticoagulation indication			
Atrial fibrillation	-0.286 (0.326)	-0.878	0.38
Pulmonary embolism	0.060 (0.371)	0.161	0.87
Deep vein thrombosis	-0.009 (0.302)	-0.029	0.98
Heart valve prosthesis	-0.273 (0.370)	-0.737	0.46

SE, standard error; VKA, vitamin K antagonists; TIA, transient ischemic attack.
